# Using visual feedback distortion to alter coordinated pinching patterns for robotic rehabilitation

**DOI:** 10.1186/1743-0003-4-17

**Published:** 2007-05-30

**Authors:** Yoky Matsuoka, Bambi R Brewer, Roberta L Klatzky

**Affiliations:** 1 The Robotics Institute, Carnegie Mellon University, Pittsburgh, PA 15213, USA; 2 Mechanical Engineering, Carnegie Mellon University, Pittsburgh, PA 15213, USA; 3 Psychology Department, Carnegie Mellon University, Pittsburgh, PA 15213, USA

## Abstract

**Background:**

It is common for individuals with chronic disabilities to continue using the compensatory movement coordination due to entrenched habits, increased perception of task difficulty, or personality variables such as low self-efficacy or a fear of failure. Following our previous work using feedback distortion in a virtual rehabilitation environment to increase strength and range of motion, we address the use of visual feedback distortion environment to alter movement coordination patterns.

**Methods:**

Fifty-one able-bodied subjects participated in the study. During the experiment, each subject learned to move their index finger and thumb in a particular target pattern while receiving visual feedback. Visual distortion was implemented as a magnification of the error between the thumb and/or index finger position and the desired position. The error reduction profile and the effect of distortion were analyzed by comparing the mean total absolute error and a normalized error that measured performance improvement for each subject as a proportion of the baseline error.

**Results:**

The results of the study showed that (1) different coordination pattern could be trained with visual feedback and have the new pattern transferred to trials without visual feedback, (2) distorting individual finger at a time allowed different error reduction profile from the controls, and (3) overall learning was not sped up by distorting individual fingers.

**Conclusion:**

It is important that robotic rehabilitation incorporates multi-limb or finger coordination tasks that are important for activities of daily life in the near future. This study marks the first investigation on multi-finger coordination tasks under visual feedback manipulation.

## Background

Stroke and other neurological disorders affect more and more people as the general population ages. Early rehabilitation increases the chance that patients retain the ability to function in activities of daily life (ADL). To assist this functional gain, patients are often taught compensatory movements that are functionally effective for the level of disability they have at the time of rehabilitation. However, even if these patients regain additional movements over time, it is common for them to continue using the compensatory movements taught due to entrenched habits[1], increased perception of task difficulty[2], or personality variables such as low self-efficacy or a fear of failure[3,4]. If there is a technique to allow these patients to move away from the entrenched compensatory movements so that other potentially more effective movements can be explored and practiced, it may lead to increased function in ADL. We hypothesize that this type of exploration may be possible using virtual rehabilitation environments that can distort visual feedback of patients' movements away from their current habit without their awareness.

To date virtual and robotic rehabilitation environments have focused on increasing strength and range of motion of a single limb [1-6]. Patton et al. (2001) used the perturbation force profile to strengthen muscles and extend the range of motion. Our group has shown that visual feedback distortion in a virtual environment enables both able-bodied and traumatic brain injury (TBI) subjects to produce more force and move further than their perceived movements[4,7]. The distortion is remapping between the actual movements and the virtual visual feedback the subjects receive about their movements. The Just Noticeable Difference (JND) defines the lowest amount of discrepancy between the actual movements and the virtual visual feedback. As long as the distortion is less than the JND, the subjects are unable to detect the distortion[4,8]. Subsequently, we showed that young, elderly, and disabled subjects were able to increase force and distance moved, without perceiving the difference and without an increased amount of perceived effort[4,9]. When this feedback distortion environment was used for rehabilitation, we witnessed a long-term increase of the maximum force production and range of motion of stroke and chronic TBI subjects[4,6].

It is important that robotic rehabilitation incorporates multi-limb or finger coordination tasks that are important for ADL in the near future (note: in this paper, the thumb is also called a finger for simplicity). None of the previous research in robotic or virtual rehabilitation to our knowledge, however, addresses coordination tasks. The purpose of the visual feedback distortion for individual finger training was to change the force or distance goal corresponding to each level, thus challenging the maximum force production or range of motion. In the multi-finger case, distorting visual feedback of all fingers equally does not result in the overall change in coordination. The present work used a different distortion paradigm that we call "error enhancement" on individual fingers. According to this paradigm, subjects were asked to work at a demanding task with an objective performance criterion. Departures from ideal performance were displayed as errors, which were distorted to appear larger. Some evidence for the utility of this approach for one limb comes from Wei et al[10], who showed that magnifying visual error by physically displacing the arm's trajectory resulted in smoother and straighter trajectories.

In this paper, we investigated whether we could train able-bodied individuals to achieve a prescribed pinching movement with distort visual feedback of individual fingers consisting of the error relative to the desired movement. Specifically, we aimed to answer (1) whether different coordination pattern could be trained with visual feedback and have the learned pattern transfer to trials without visual feedback, (2) whether distorting individual finger at a time would affect the amount of error reduction for both fingers, and (3) whether overall learning would be sped up by distorting individual fingers.

## Methods

### Experimental setup

#### Physical setup

**Figure 1:**
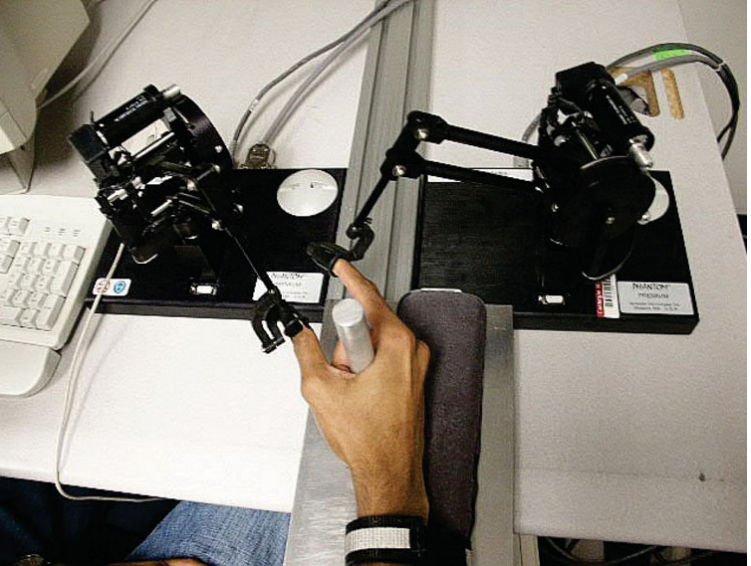
Two PHANTOM™ robots were used to track and distort the index finger and thumb movement trajectories separately.

The robotic environment used in this experiment is shown in Figure 1. One or two Premium 1.0 PHANTOM™ force-feedback robots (SensAble Technologies, Inc., Woburn, MA) were used. Each robot has 3 active degrees of freedom and a position resolution of 0.03 mm[11]. The standard finger cuff provided by Sensable Technologies provided additional 3 passive degrees of freedom at the fingertip. For the conditions involved two fingers, the subject placed the index finger in one finger cuff and the thumb in the other. For the conditions involved only the thumb, the subject placed only the thumb in a robot. The other fingers grasped a post to keep the hand stationary throughout the experiment. The subject sat with the arm flexed at the elbow and the forearm horizontal.

#### Calibration setup

Once the pinching movement had been isolated from the rest of the hand and arm movements, the experimenter moved the subject's index finger and thumb back and forth starting from the widest pinch (100% calibrated pinch span) and ending with their fingers touching each other (0% calibrated pinch span) for calibration. The experimenter moved the fingers to assure that both fingers span enough distance during this calibration. For the thumb-only conditions, the experimenter moved the thumb alone from 100% to 0% of the pinching span. While subject's fingers were moved back and forth, fingers' mean pinch span limits and trajectory were calculated.

#### Visual feedback setup

**Figure 2:**
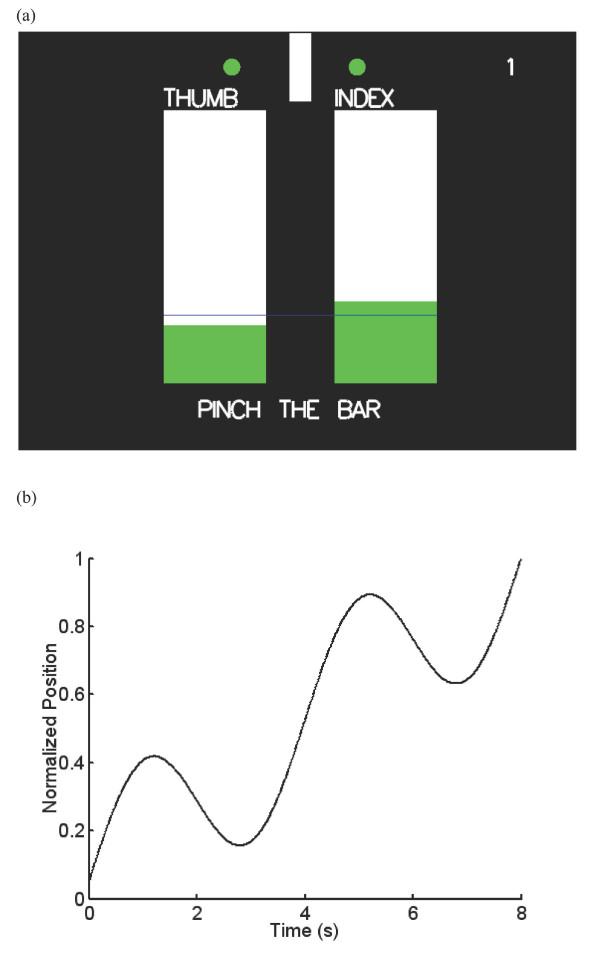
(a) The subjects' task was to pinch a virtual object displayed on the top of the screen while they observed the normalized distance traveled displayed as a bar graph. (b) For each trial, the target line across both bars moved from the bottom to the top in the prescribed manner.

In the trials, the finger movements were designed to start from 80% of their calibrated pinch span (virtual walls were placed to constrain fingers). Then a virtual object (width of 26 mm) with hard virtual boundary was placed at the center of 0% calibrated pinch location. The distance between the 80% of the pinching span and the surface of the object for both fingers was assigned to be the full motion during the experiment, and the distance between these points for both fingers was normalized and displayed as two bar graphs on the computer screen (Figure 2(a)). Bottom of the bar represented the widest pinch pose, and the top of the bar represented the surface of the object. The shaded area of the bar graph moved up as the fingers moved toward the virtual object. In addition to the normalized distance of the finger movements, the stationary virtual object (white rectangle) and the finger movements used to pinch the object (circles to the left and right of the rectangles) were displayed at the top of the screen (Figure 2(a)). For the conditions requiring only the thumb motion, the display was exactly the same except there was only one bar indicating the location of the thumb.

A target line crossing both bars moved up and down to indicate the desired finger movement trajectory. The line started at the bottom of the bars, and as soon as the subject crossed the line with the shaded portion of either bar, the line began to move. On every trial, the line moved for 8 seconds according to the equation L=0.5+0.95Δt8+0.2375sin⁡(4πΔt8)
 MathType@MTEF@5@5@+=feaafiart1ev1aaatCvAUfKttLearuWrP9MDH5MBPbIqV92AaeXatLxBI9gBaebbnrfifHhDYfgasaacH8akY=wiFfYdH8Gipec8Eeeu0xXdbba9frFj0=OqFfea0dXdd9vqai=hGuQ8kuc9pgc9s8qqaq=dirpe0xb9q8qiLsFr0=vr0=vr0dc8meaabaqaciaacaGaaeqabaqabeGadaaakeaacqWGmbatcqGH9aqpcqaIWaamcqGGUaGlcqaI1aqncqGHRaWkcqaIWaamcqGGUaGlcqaI5aqocqaI1aqndaWcaaqaaiabfs5aejabdsha0bqaaiabiIda4aaacqGHRaWkcqaIWaamcqGGUaGlcqaIYaGmcqaIZaWmcqaI3aWncqaI1aqncyGGZbWCcqGGPbqAcqGGUbGBdaqadaqaamaalaaabaGaeGinaqdcciGae8hWdaNaeuiLdqKaemiDaqhabaGaeGioaGdaaaGaayjkaiaawMcaaaaa@4D12@ where Δ*t *is the time in seconds since the beginning of the trial and *L *is the normalized position of the line along the bars (Figure 2(b)). This specific movement profile was chosen as a challenging but learnable coordination pattern so that we can observe a learning trend over tens of trials.

#### Trials without distortion

Trials without distortion provided the true normalized finger movements as the visual feedback. The trial absolute error (the mean absolute difference between the normalized position of each finger and the target line) was displayed to the subject after each trial.

#### Trials with distortion

Trials with distortion had a magnification of the error between the finger position and the position of the target line by 20%. This distorted visual feedback was provided in real time and the numerical error given to the subject at the end of each trial was also increased by 20%.

#### Trials with no-feedback

After the first 20 trials, one no-feedback trial was incorporated randomly every 20 trials. On the no-feedback trials, the normalized position of each finger was not shown; the screen appeared the same, except that no part of the visual feedback bars was shaded. These trials were included to assess whether subjects could reproduce the target movement without feedback about finger position.

### Experimental procedure

Fifty-one subjects between 18 and 35 years of age participated in the experiment. Table 1 shows five experimental conditions of ten subjects each (except for one condition containing eleven subjects), and subjects were assigned to the conditions randomly to assure gender and age balance. No subject participated in more than one condition. All gave informed consent and performed the experiment with the dominant right hand. No subject had a known history of neurological injury.

**Table 1:** Three experimental conditions. Finger that received visual feedback distortion is listed for five conditions tested.

Condition (Acronym)	Number of Subjects	Distorted Finger(s)		
		81–120	121–160	161–200

ITB	11	Index	Thumb	Both
TIB	10	Thumb	Index	Both
C	10	None	None	None
NTN	10	None	Thumb	N/A
TNN	10	Thumb	None	N/A

#### ITB (index-thumb-both) condition

The ITB condition (along with TIB and C below) was designed to address (1) whether different coordination pattern could be trained with visual feedback and have the learned pattern transfer to trials without visual feedback, (2) whether distorting individual finger at a time would affect the amount of error reduction for both fingers, and (3) whether overall learning would be sped up by distorting individual fingers.

This condition consisted of 200 trials with the first 80 trials designated for establishing baseline without distortion. The subject then encountered a section of 40 trials (trials 81–120) in which the visual feedback for the index finger was distorted, followed by a section of 40 trials (trials 121–160) in which the visual feedback for the thumb was distorted. The experiment concluded with a section of 40 trials (trials 161–200) in which the visual feedback for both the index finger and the thumb was distorted.

#### TIB (thumb-index-both) condition

The procedure for the TIB condition was similar to the ITB procedure, except that the section of trials with distorted thumb feedback occurred before the section of trials with distorted index finger feedback. This condition was included to capture the effect of the thumb being distorted as the first distorted finger (in ITB, we can only observe the effect of thumb distortion after the thumb performance was influenced by the index finger distortion), and to compare whether there is any overall learning difference by distorting the thumb first in stead of the index finger.

#### C (control) condition

In the C condition, all 200 trials contained no distortion. This condition acted as the controls for ITB and TIB conditions to show whether the individual finger distortion changed the shape of error reduction profile and whether the overall learning could be sped up.

#### NTN (thumb-only condition mirroring ITB) condition

In ITB, TIB, and C conditions, subjects learned to move two fingers at the same time. To understand whether and/or how much of the change in the shape and speed of error reduction came from the fact that subjects were learning two finger motions at the same time, we conducted a similar experiment as the ITB condition without the index finger movement.

This condition consisted of 160 trials with the first 80 trials designated for establishing baseline without distortion. To mimic the ITB condition, the subject then encountered additional 40 trials (trials 81–120) without distortion (for the ITB, the index finger was distorted during trials 81–120). Then the following 40 trials (trials 121–160) had distortion in the visual feedback for the thumb.

#### TNN (thumb-only condition mirroring TIB) condition

The procedure for the TNN condition was similar to the NTN procedure, except it mirrored the TIB condition. Therefore, the subject encountered thumb distortion during trials 81–120, and no distortion during trials 121–160.

#### Questionnaires

Post-experiment questionnaires were provided to assess whether the subjects detected distortion, whether they used specific movement strategies, and their perception of task difficulty.

### Data analysis

For data analysis, the experiments are divided into blocks of 20 trials. First 20 trials are considered to be practice and Block 1 corresponds to trials 21 to 40 and so forth until Block 9 which corresponds to trials 181 to 200. There were four sections corresponding to trials 41–80 (Section 1), trials 81–120 (Section 2), trials 121–160 (Section 3), and trials 161–200 (Section 4). These sections were typically under different distortion types (except for the control condition).

We define a few different measures to analyze the effect of the different distortion modes. *Trial absolute error *is the mean absolute difference between the normalized position of each finger and the goal finger location. *Mean absolute error *is the sum of the trial absolute error for each block or section for each subject. *Normalized error *is the mean absolute error divided by the mean absolute error for the first block/section to remove the inter-subject variability in baseline performance. *Mean absolute difference *is computed over trials and over subjects between the normalized position of the index finger and the normalized position of the thumb. All four measures are unit-less. *Mean lag *(in seconds) is a mean computed over trials, fingers and subjects of the difference in time that maximized the correlation between the finger position and target line position for each trial and finger. Essentially, this quantity measures the time period by which the subject's response trailed the target movement.

A contrast analysis was conducted when the distortion switched from one finger to another to test whether the crossover trend between the increasing error in one finger and the decreasing error in another finger was significant. Repeated-measures ANOVAs were conducted for index finger and the thumb. Test condition was a between subjects factor, and section was a within-subjects factor.

## Results

### Different coordination patterns were learned

**Figure 3:**
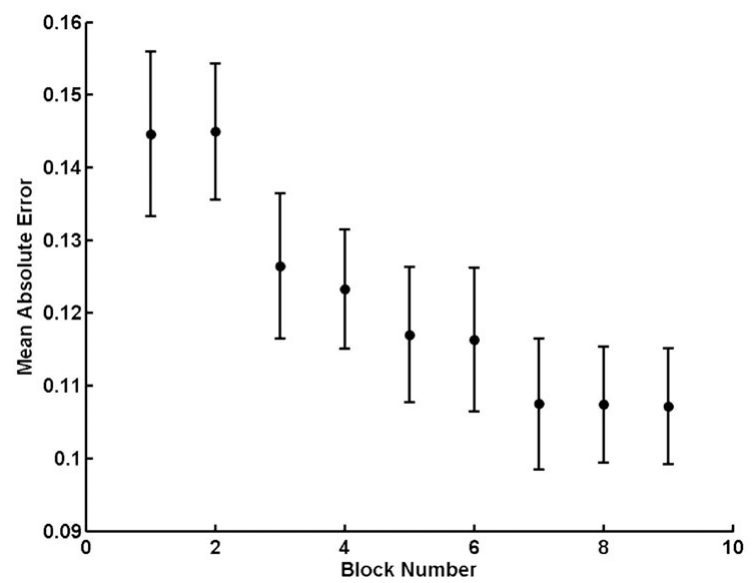
Learning over time for C condition. A decrease in the total mean absolute error as a function of block number occurred as the experiment progressed.

The change in mean absolute error over time in the C condition is shown in Figure 3. Data from the no-feedback trials were excluded from this analysis. The mean total absolute error for Block 1 was significantly different from that for Block 9 (*p *< 0.001). When the mean total absolute error for the index finger was compared to that of the thumb, the thumb error was significantly larger for Block 1 (*p *= 0.007), but not for Block 9 (*p *= .13). The mean absolute difference was less for the latter trials (*p *< 0.001), which means that subjects learned to move the thumb and the index finger in a more coordinated fashion during the experiment.

**Figure 4:**
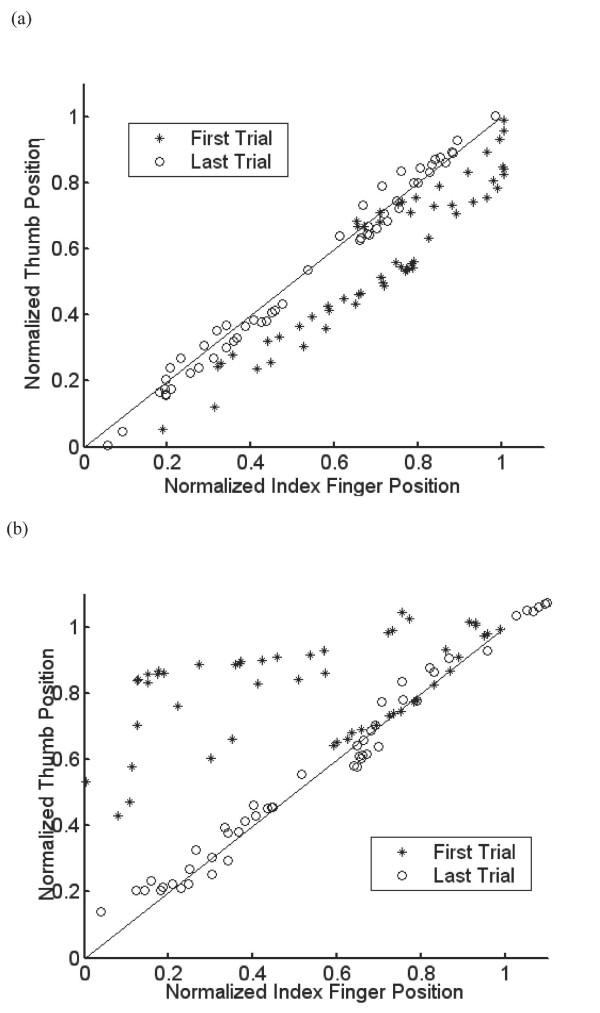
The pinching movement pattern recorded in the first trial (*) was different from the learned pinching movement recorded in the last trial (o). (a) Some subjects led with their index finger first (trial 1 is denoted with '*') and learned to move their fingers in a prescribed manner by the end of the experiment (trial 200 is denoted with 'o'). (b) Others led with their thumb.

**Figure 5:**
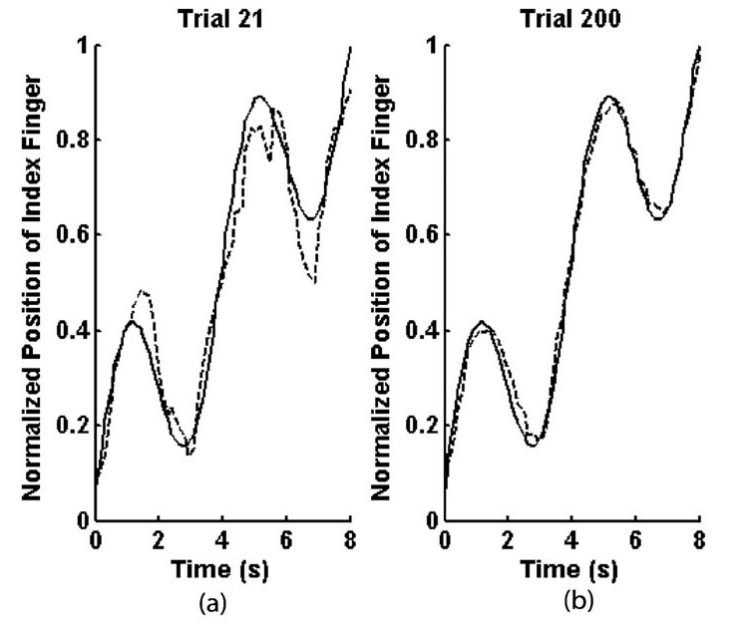
(a) Performance of a single subject on trial 21. The solid line represents the normalized position of the target line as a function of time, and the dashed line represents the normalized position of the index finger. (b) Performance of the same subject on trial 200. The subject has reduced the error of the index finger relative to the target line, but the lag between the target line and the path of the index finger has remained approximately the same.

Figure 4 shows the time evolution of one pinch movement in a normalized fashion. Subjects led with one of the fingers at first, and learned to move both fingers together by keeping the same normalized distance (denoted as the diagonal line on Figure 4) over time. Some subjects led with their index finger first (trial 1 is denoted with '*') and learned to move their fingers in a prescribed manner by the end of the experiment (trial 200 is denoted with 'o') as shown in Figure 4A. And others led with their thumb (Figure 4B). Subjects learned to keep the normalized position of each finger closer to the target line during the experiment, but the mean lag of each finger remained the same (Figure 5). The mean lag for Block 1 was not significantly different from that for Block 9 for either the index finger or the thumb (*p *= 0.78 for index, 0.50 for thumb).

**Figure 6:**
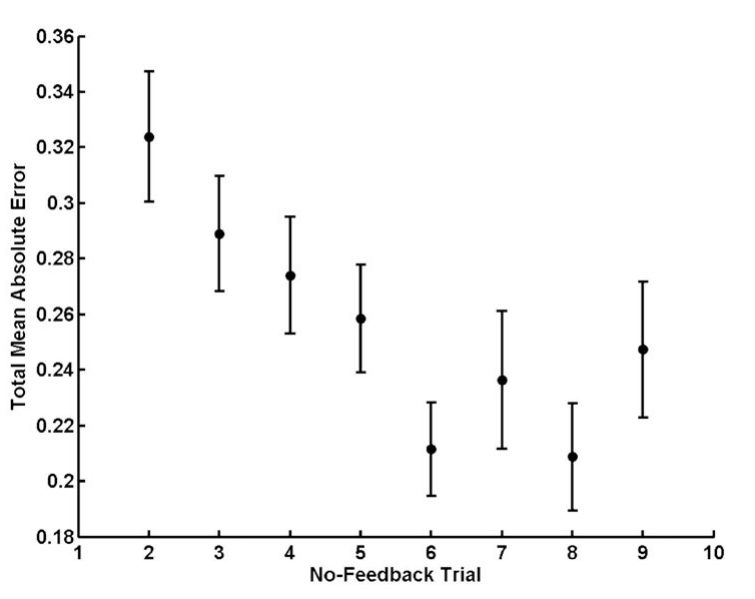
Results of the no-feedback trials for C condition. Data from the first no-feedback trial were excluded. The total mean absolute error was significantly larger for no-feedback trials than for the with-feedback trials, but a decrease in total absolute error and an increase in coordination did occur during the experiment for the no-feedback trials.

Figure 6 shows the total mean absolute error for the no-feedback trials to examine whether learning in the with-feedback trials transferred to improvements in performance on the no-feedback trials. The first no-feedback trial was excluded because despite instructions, many subjects did not execute the task when the first no-feedback trial occurred. The difference between the mean total absolute error on the second and last no-feedback trials was significant (*p *= 0.05). The mean absolute difference decreased from the second no-feedback trial to the last (*p *= 0.02), showing an improvement in coordination of the finger and thumb on the no-feedback trials. Subjects had significantly larger errors on the no-feedback trials than that for the with-feedback trials (*p *< 0.001).

### Distortion effect on error reduction

**Figure 7:**
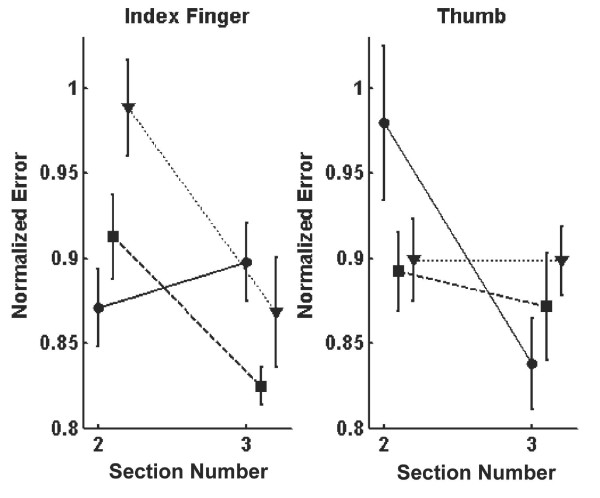
The effects of distortion on learning of the coordination task. Squares represent the C condition, circles represent the ITB condition, and triangles represent the TIB condition. (a) The normalized error for the index finger. The change from section 2 to 3 for the ITB condition is significantly different from that of the controls. (b) The normalized error for the thumb. Again, the change from section 2 to 3 for controls was significantly different from that for subjects in the ITB condition. No differences were found between control and TIB subjects.

The data from ITB, TIB and C conditions are assessed to observe the effects of the distortion. Figure 7 shows the normalized error for sections 2 and 3 for the index finger (Figure 7A) and the thumb (Figure 7B). The normalized index finger error for section 2 did not differ significantly for the ITB and C conditions (*p *= 0.24) but differed significantly between the TIB and C conditions (*p *< 0.001). For section 3, the normalized index finger error was significantly different for the ITB and C conditions (*p *= 0.01). The normalized thumb error for the ITB or TIB condition did not differ from the C condition for either section.

When the thumb was distorted in section 3 for the ITB condition, the error reduction for the thumb was significantly more than for the C condition (*p *= 0.005). Meanwhile, the error reduction for the index finger error was significantly less than for the C condition (*p *= 0.003). The contrast analysis showed the contrast of 0.17 for the ITB condition and -0.063 (negative contrast means the index finger dropped more than the thumb) for the C condition, and the crossover trend was significant for both conditions (*p *< 0.001 for ITB and *p *= 0.003 for C).

Repeated-measures ANOVA for the index finger showed no significant main effect of conditions (*p *= 0.21), significant main effect of section (*p *< 0.001), and significant interaction of condition and section (*p *< 0.001). Repeated-measures ANOVA for the thumb showed no significant main effect of conditions (*p *= 0.83), significant main effect of section (*p *< 0.001) and significant interaction of condition and section (*p *< 0.001). The main effect of section was due to learning done after trial 80, since trials before that weren't included in the analysis.

Data from NTN and TNN conditions were analyzed with ANOVA for between-subjects factor of condition and within-subjects factor of section. No significant main effect of subject condition was observed (*p *= 0.58). No significant main effect of section was observed (*p *= 0.23) indicating no learning past trial 80 since this was a simpler task than the two finger coordination tasks (such as ITB, TIB and C). And finally there was no significant interaction between the TNN and NTN conditions (*p *= 0.99) showing the order of distortion didn't change the results.

### Distortion did not change the final performance

To determine whether distortion affected terminal performance, we compared the mean normalized error in section 4 for each distortion condition to the C condition. None of these comparisons was significant (ITB: *p *= 0.07 for index, 0.69 for thumb; TIB: *p *= 0.14 for index finger, 0.99 for thumb).

Post-experiment questionnaires revealed that none of the subjects detected the distortion. All but four subjects stated that they tried to move the finger and the thumb in a coordinated way. And all subjects stated that the task required a significant amount of concentration and felt that the task was extremely difficult.

## Discussion

Altering the coordination pattern among multiple fingers is considered to be a challenging problem. Latash et al[12] conducted an experiment where subjects ramped up the sum of the static forces produced by all their fingers shown on the computer screen from zero to a designated force. When subjects were informed of the coefficients used for the fingers (i.e. some finger forces were multiplied by 0.5 or 2 before being summed), subjects immediately changed their force production pattern to compensate for those changes. When subjects were not informed of the coefficients, no adaptation to the distorted feedback was observed and they used the same coordination pattern as when all the coefficients were 1. This result shows the difficulty in changing the coordination pattern among multiple fingers. It is, however, interesting to note that our results showed the change in the coordination pattern between the index finger and the thumb carried over to the no-feedback trials.

For all subjects in our experiment, the habitual pinching pattern was not symmetric between the index finger and the thumb. Despite their different habitual coordination patterns, we were able to train subjects to use the same new coordination pattern with visual feedback guidance. This learning was confirmed to have taken place even without the presence of the visual feedback during the interleaved trials with no feedback. In this task, subjects learned a particular pattern of movement as they tried to minimize visual error. Because emphasis was placed on the visual error, it is no surprise that a subject's error was greater when executing the learned movement without visual feedback of position. However, the mean total absolute error in the no-feedback case still decreased as the experiment progressed. More transfer to the no-feedback case might have been observed if more no-feedback trials had been included. For effective learning and transfer of a motor task to occur, subjects may need to learn to use internal cues rather than relying on extrinsic feedback[13,14].

Although the TIB condition more closely mimicked the C condition, they too showed a steep decline in index finger error and a counter-learning trend in thumb error when distortion shifted from the thumb to the index finger (section 2 to section 3). The lack of significant differences between TIB and C conditions can be explained by the fact that the trial absolute error for the thumb was significantly larger than that of the index finger for the C condition at the beginning of the experiment. Both C and TIB subjects saw the thumb error as larger in section 2, and both conditions focused on minimizing that error. The thumb error was not significantly different from the index finger error for the C condition at the end of the experiment. Thus, as the experiment proceeded, subjects in the C condition may have worked on error reduction for both fingers more evenly. This may parallel the focus of TIB subjects to the index finger error and then to both fingers.

It is interesting to note that there was a trend for the non-distorted finger's error to increase. It is possible that the subjects were unable to maintain the performance of the non-distorted finger while they were concentrating on reducing the error of the distorted finger. This view is consistent with the idea of minimizing variance in task space while the motor variability appears in the uncontrolled manifold[15]. This implies that the focus was originally divided between two fingers to achieve two tasks at the same time. When the distortion emphasized the error of one of the fingers, the task requirement for that finger went up, and the amount of task-level focus that could be provided for the non-distorted finger decreased. This is consistent with the fact that all of the subjects indicated in the questionnaire that the task was extremely difficult and that it required a high level of concentration at all times. If the task is made easier, it may be possible to devote more focus on the distorted finger and reduce the error, while not compromising the non-distorted finger task. In addition, if the distortion changes the task goal (as in our previous studies and therapeutic paradigms) rather than enhancing on the error, the result may be different. This is a key issue that must be addressed, in order to investigate and retrain movement coordination patterns.

The TIB and ITB conditions did not perform better than the C condition when both finger movements were distorted together. There was no difference in performance between the index finger and the thumb because they were treated identically. This fact shows that simply exaggerating the error for both fingers together is not effective. Also, in the TNN and NTN conditions, distortion had no effect on the normalized error of subjects. These results are similar to those of Patton[16], which reported that error augmentation using a multiplicative gain did not improve terminal performance in a reaching task. Error augmentation through a constant offset was found to be more effective[16], but that type of distortion would not be relevant for the task we considered.

It is a common fear that the learned effect in a distorted environment would "wash out" to the baseline immediately after the training took place. However, when working with disabled individuals, these effects have been shown to not wash out (unlike for able-bodied individuals)[3,6]. Learned effects do not wash out for disabled individuals because they may have learned to activate different sets of muscles during the distorted feedback training. When the distorted feedback is removed, they are left with the new coordination patterns that they practiced repetitively during therapy, and which work to accomplish tasks in daily life.

## Conclusion

Our ultimate goal is to manipulate visual feedback in a virtual robotic rehabilitation environment to steer patients away from entrenched coordination habit that may not be best for them (e.g. either because they have the muscular strength to improve on task performance, or because this habitual movement is causing other physical problems such as tendonitis). We showed that (1) training under visual feedback allowed new coordination pattern to transfer to no-feedback trials, (2) feedback distortion changed the amount of error reduced for each finger separately, and (3) distorting individual fingers separately (or together) did not affect the overall speed of learning in movement error reduction. By interleaving no visual feedback trials more often, a dependence on the visual display may be avoided and may allow better transfer of the new coordination strategy to daily activities. This study was not conducted to show that impaired or unimpaired people would be able to achieve "better" performance when the visual feedback manipulation was provided. Rather, we initiated an important investigation on multi-limb coordination tasks under visual feedback manipulation.

## Competing interests

The author(s) declare that they have no competing interests.

## Authors' contributions

YM conceived the study, provided expert guidance on experimental design and data analysis, and drafted the manuscript. BB recruited subjects, setup experiments, managed data collections, conducted data analysis, drafted a conference version of this manuscript, and helped edit the manuscript. RK provided expert guidance on experimental design, statistical analysis, and helped edit the manuscript. All authors approved the manuscript.
